# Peculiar Cat with Many Lives: PUMA in Viral Infections

**DOI:** 10.3390/cells15030278

**Published:** 2026-02-01

**Authors:** Zbigniew Wyżewski, Justyna Stępkowska, Pola Pruchniak, Adrianna Niedzielska, Karolina Paulina Gregorczyk-Zboroch, Matylda Barbara Mielcarska

**Affiliations:** 1 Institute of Biological Sciences, Cardinal Stefan Wyszynski University in Warsaw, Dewajtis 5, 01-815 Warsaw, Poland; 2 Institute of Family Sciences, Cardinal Stefan Wyszynski University in Warsaw, Dewajtis 5, 01-815 Warsaw, Poland; j.stepkowska@uksw.edu.pl; 3 Division of Immunology, Department of Preclinical Sciences, Institute of Veterinary Medicine, Warsaw University of Life Sciences—SGGW, Ciszewskiego 8, 02-786 Warsaw, Poland; pola_pruchniak@sggw.edu.pl (P.P.); adrianna_niedzielska@sggw.edu.pl (A.N.); karolina_gregorczyk-zboroch@sggw.edu.pl (K.P.G.-Z.); matylda_mielcarska@sggw.edu.pl (M.B.M.)

**Keywords:** Bcl-2 family, apoptosis, mitochondria, PUMA, HSV-1, EBV, HBV, HIV-1, IAV, DENV

## Abstract

Apoptosis is a natural mechanism that shapes morphogenesis and helps maintain tissue homeostasis in healthy organisms. It is also extensively studied in the context of pathologies such as cancer and viral infections. The course of the latter strictly depends on host cell viability; therefore, regulators of apoptosis may play essential roles in distinct viral infections as well as virus-dependent diseases. The p53-upregulated modulator of apoptosis (PUMA), a pro-apoptotic member of the B-cell lymphoma 2 (Bcl-2) family, directly disrupts mitochondrial integrity, thereby promoting the intrinsic apoptotic pathway. PUMA-mediated cell death act as a double-edged sword that may either facilitate viral infection and its consequences or counteract them, depending on the infectious agent and the complex context of pathogen–host interactions. Accordingly, various viruses have evolved strategies to modulate host cell viability to their advantage by targeting PUMA—either by suppressing transcription of the PUMA gene, binding and inactivating the PUMA protein, or, conversely, inducing its production. In this work, we describe the role of PUMA in infections caused by distinct viruses and in associated diseases, viral strategies for modulating PUMA-related signaling pathways, and potential therapeutic implications.

## 1. Introduction

Apoptosis is a type I programmed cell death (PCD I) mechanism that does not trigger inflammation and is crucial in maintaining organismal homeostasis [1]. It is essential for proper embryonic development, cellular turnover within tissues, and the proper functioning of the immune system. Moreover, apoptosis contributes to host defense mechanisms by eliminating damaged or pathogen-infected cells [2]. Apart from PCD I, additional forms of PCD have been described, including autophagy, necroptosis, pyroptosis, PANoptosis, ferroptosis, and cuproptosis [3,4,5,6,7,8,9,10,11,12,13].

Apoptosis proceeds according to a strictly defined sequence of events and is accompanied by characteristic morphological changes in the cell [14,15]. The molecular mechanism of PCD I is highly complex, involving numerous proteins and signaling molecules that govern both the initiation and execution of the process. Two major apoptotic pathways are distinguished: extrinsic and intrinsic [16]. An illustration of the key components of both the extrinsic and intrinsic apoptotic pathways is provided in Figure 1 of Wyżewski et al. [17].

The extrinsic apoptotic pathway is triggered by the activation of death receptors of the tumor necrosis factor receptor superfamily, such as tumor necrosis factor (TNF) receptor 1 (TNFR-1) and Fas [18,19], upon binding of their respective ligands. Ligand-induced receptor trimerization enables recruitment of adaptor proteins, including Fas-associated death domain (FADD) and TNF receptor-associated death domain (TRADD) [20], leading to activation of initiator caspases, primarily caspase-8 or caspase-10, within the death-inducing signaling complex (DISC)) [21]. This signaling cascade culminates in the activation of effector caspases, mainly caspase-3 and caspase-7, executing apoptosis [22,23,24,25,26]. The intrinsic (mitochondrial) apoptotic pathway is activated by intracellular stress signals, including DNA damage, hypoxia, viral infections, reactive oxygen species (ROS), and nutrient or cytokine deprivation. These stimuli trigger mitochondrial dysfunction, characterized by increased outer membrane permeability and a loss of membrane potential, ultimately leading to apoptosis [27]. Regulation of this pathway is mediated primarily by the B-cell lymphoma 2 (Bcl-2) protein family, whose activity is tightly controlled by the tumor suppressor p53 [28]. Upon stress, p53 promotes activation and oligomerization of the pro-apoptotic proteins, Bcl-2-associated X protein (Bax) and Bcl-2 homologous antagonist/killer (Bak), at the mitochondrial outer membrane (MOM), resulting in MOM permeabilization (MOMP) [29]. MOMP leads to the release of mitochondrial apoptogenic factors, such as cytochrome c, which activates the caspase cascade via apoptosome formation, as well as caspase-independent effectors including apoptosis-inducing factor (AIF) [29,30,31,32].

The key transcription factor that integrates the response to DNA damage with cell cycle control mechanisms and participates in both apoptotic pathways is p53 [33]. Under physiological conditions, the level of p53 remains low due to strict regulation by its negative regulators [34,35]. Upon exposure to intrinsic or extrinsic stress, such as DNA damage, hypoxia, nutrient deprivation, or oncogenic signals, ubiquitination of p53 is inhibited, leading to a rapid increase in its intracellular concentration. Accumulated p53 is subsequently activated and stabilized by various post-translational modifications [36,37,38,39]. Stabilized nuclear p53 binds to target DNA and regulates the transcription of multiple genes, thereby initiating signaling cascades that result in cell cycle arrest, DNA repair, or apoptosis [40,41,42,43,44].

The p53-upregulated modulator of apoptosis (PUMA) is a key mediator of the cytoplasmic pro-apoptotic functions of p53. As a BH3-only member of the Bcl-2 family, PUMA contains a BH3 domain that facilitates interaction with other family members [45,46]. Importantly, among all BH3-only proteins, only PUMA has been shown to release p53 from its inhibitory complex by directly binding B-cell lymphoma-extra large (Bcl-xL) protein [47]. While cytoplasmic p53 can be sequestered and inactivated by Bcl-xL [47,48], nuclear p53 induces the transcription of multiple pro-apoptotic genes, including PUMA itself [45,49]. Once expressed, PUMA strongly binds to and neutralizes anti-apoptotic members of the Bcl-2 family [50]. Moreover, upon release from Bcl-xL, cytoplasmic p53 can directly activate Bax or Bak, thereby initiating MOMP or apoptosis [43].

Apoptosis is one of the key mechanisms limiting viral replication in infected host cells. However, since viable cells are required for genome replication and progeny virion production, viruses have developed mechanisms to prevent host cell death effectively [51,52]. Viral proteins can both inhibit and induce the apoptotic process. Most often, however, viruses block or delay apoptosis, ensuring sufficient time for genome replication and assembly of progeny virions. Such regulation may occur during lytic and latent infections: by preventing premature cell death, which allows increased virion production [51], or by supporting cell survival during host cell transformation [53].

Viruses modulate apoptosis by interfering with both the extrinsic and intrinsic pathways. DNA viruses encode inhibitors that can block the extrinsic apoptotic pathway by suppressing the activity of cellular FLICE-like inhibitory protein (c-FLIP), which binds to FADD, or by neutralizing death ligands that promote caspase-8 activation [27]. In contrast, viral proteins capable of inhibiting the intrinsic pathway act by mimicking the function of host anti-apoptotic proteins or by neutralizing pro-apoptotic members of the Bcl-2 family, including Bax [54].

Some viruses adopt the opposite strategy and actively induce apoptosis. In this way, they exploit apoptotic bodies as “transmission vehicles”, facilitating the release and spread of progeny virions while avoiding recognition by the host immune system [51,55]. A notable example of a virus that induces apoptosis is the Zika virus (ZIKV), which directly impacts the intrinsic apoptotic pathway by modulating the recruitment and activation of Bax [56].

On the whole, both forms of apoptosis modulation, its inhibition and induction, can adversely affect the host organism. Inhibition of apoptosis promotes viral replication and hinders the clearance of infection, whereas induction of apoptosis may result in tissue and organ damage due to excessive cell death [57].

This article aims to provide a comprehensive overview of the role of PUMA as a critical regulator of apoptosis in the context of viral infection. By highlighting the molecular mechanisms of PUMA activity and its ability to shape host–virus interactions, this review seeks to clarify the nature of PUMA’s contribution to infection outcomes and to assess its potential relevance as a therapeutic target.

While several review articles have addressed the role of PUMA in apoptosis and cancer or have broadly described apoptotic molecular pathways during viral infections, a focused synthesis of PUMA-centered mechanisms in this context is still lacking. Notably, the existing virological review literature typically presents PUMA as one of many BH3-only proteins or Bcl-2 family members, rather than providing a dedicated and comprehensive analysis specifically concentrated on this molecule. In this work, we provide a comparative overview of the distinct effects exerted on PUMA by a broad spectrum of viral infections, which either promote or suppress the PUMA-mediated apoptotic cascade. By linking these processes to pathogen-driven pathologies and antiviral therapeutic strategies, our review offers a novel perspective on PUMA as a central regulatory hub in virus–host interactions.

## 2. Literature Search and Selection Criteria

Literature for this narrative review was retrieved primarily from the PubMed, Scopus, and Web of Science databases. Searches were conducted up to September 2024, with an emphasis on studies published within the last 5–15 years. Combinations of the keywords “PUMA” and “viral infection”, “virus”, or “apoptosis” were used, together with either the full or abbreviated names of individual viruses discussed in this work. Original research articles and relevant review papers published in English were considered. Priority was given to studies providing mechanistic insights into PUMA regulation or function during viral infection. Additional references were identified through manual screening of cited literature.

## 3. Molecular Biology of PUMA

As previously described, PUMA is a member of the Bcl-2 protein family and belongs to the BH3-only subgroup. It contains a characteristic BH3 domain that enables interaction with other members of the Bcl-2 family [45,46]. The *PUMA* gene encodes four isoforms: α, β, γ, and δ, of which only PUMA-α and PUMA-β possess the BH3 domain [45]. The PUMA protein consists of 193 amino acids and comprises two functional domains. The BH3 domain is amphipathic and adopts an α-helical structure, while the hydrophobic segment localized in the C-terminal region directs PUMA to mitochondria, functioning as a mitochondrial localization signal (MLS) [58,59].

*PUMA* expression is generally maintained at low levels but can be induced either in a p53-dependent manner (in response to stress) or independently of p53 through other transcription factors, such as p73 and E2F1 in response to DNA damage [60,61], or through forkhead box O3 (FOXO3a) in response to cytokine or growth factor deprivation [62]. Since PUMA can bind to and inactivate all anti-apoptotic members of the Bcl-2 family, its activity must be tightly regulated [63].

p53 plays a crucial role in regulating the expression of *PUMA*. Transcriptional activation of *PUMA* requires the direct binding of nuclear p53 to specific sequences within its promoter. Studies have shown that only the binding of p53 to the *PUMA* promoter enables the initiation of transcriptional processes, such as the recruitment of transcriptional coactivators (e.g., p300) and chromatin modifications, including histone H4 acetylation. The results obtained by the researchers highlight the direct, specific, and indispensable role of p53-binding sites in the regulation of *PUMA* transcription [64].

*PUMA* expression can also be regulated in a p53-independent manner through the activity of other transcription factors. It has been demonstrated that p73 can induce *PUMA* transcription, thereby activating the apoptotic pathway involving Bax translocation to the mitochondria [61]. Similarly, the transcription factor FOXO3a can directly interact with the *PUMA* promoter, regulating its expression in response to cytokine or growth factor deprivation [62]. Another important regulator of *PUMA* is the transcription factor E2F1, which increases PUMA levels within the cell, contributing to apoptosis induction [65].

The p53-PUMA pathway is subject to complex regulation involving additional proteins that modulate its activity. It has been shown that the transcriptional repressor Slug antagonizes p53-dependent apoptosis by repressing *PUMA* expression in hematopoietic progenitor cells [66]. Another regulatory mechanism involves the interaction of PUMA with p23, a small chaperone protein with anti-apoptotic function. Prolonged endoplasmic reticulum (ER) stress induces caspase-dependent cleavage of p23, which abolishes its interaction with PUMA. As a result, PUMA becomes more available for binding to Bax, thereby facilitating the initiation of apoptosis [67].

It has been demonstrated that PUMA can directly mediate the activation of the pro-apoptotic proteins Bax and Bak [68]. The first one is stabilized by its α1 helix, which controls the engagement of the α9 helix within the dimerization pocket. PUMA transiently binds to the α1 helix of Bax, relieving its autoinhibition. This interaction induces conformational changes in Bax, exposing its N- and C-terminal ends. As a result, the C-terminal transmembrane domain becomes available for insertion into the MOM. PUMA remains associated with the N-terminally exposed Bax, thereby promoting its homo-oligomerization within the MOM. In contrast, Bak, an integral mitochondrial membrane protein, constitutively exposes its α1 helix and requires the presence of proteins such as PUMA to trigger homo-oligomerization. This process relies on the interaction between the BH3 domain of one protein and the canonical dimerization pocket of another, as evidenced by the fact that mutations within the BH1 or BH3 domains abolish the oligomerization capacity of Bax and Bak [68].

Activation of Bax/Bak may also occur indirectly, when BH3-only proteins such as PUMA bind to and neutralize anti-apoptotic members of the Bcl-2 family, including Bcl-xL. This inhibition occurs through the interaction of the amphipathic α-helix of the PUMA BH3 domain with the hydrophobic groove formed by the BH1–3 domains of anti-apoptotic proteins. This results in the inactivation of anti-apoptotic proteins and the release of pro-apoptotic Bax/Bak, enabling their activation and the induction of apoptosis [69].

The interaction between PUMA and Bcl-xL involves conserved amino acid residues within the BH3 domain and Bcl-2 family proteins. Notably, tryptophan 71 (Trp71) in PUMA is unique among BH3 domains and engages in a specific π-stacking interaction with histidine 113 (His113) in Bcl-xL. This interaction induces partial unfolding of the α2 and α3 helices in Bcl-xL, thereby abolishing its ability to bind p53. Importantly, studies have shown that substituting Trp71 with alanine prevented the release of p53 from the Bcl-xL complex [70].

Studies have shown that PUMA undergoes active proteasome-dependent degradation [63]. In particular, PUMA is subject to post-translational regulation through phosphorylation. It has been demonstrated that PUMA is phosphorylated at multiple sites, with serine 10 (Ser10) identified as the primary phosphorylation site. Researchers concluded that phosphorylation at Ser10 promotes PUMA degradation, thereby limiting its pro-apoptotic potential and supporting cell survival. These findings indicate that phosphorylation at Ser10 plays a critical role in determining the rate of PUMA turnover, as a non-phosphorylatable PUMA mutant is degraded more slowly than the wild-type protein [63].

## 4. PUMA in Viral Infections

**Figure 1:**
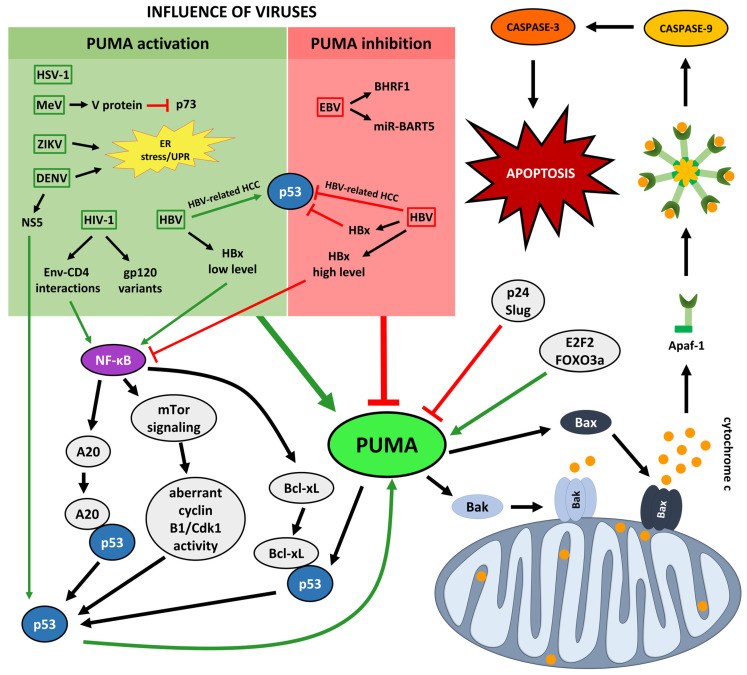
The effect of viral infections on PUMA protein. Arrow colors indicate the type of influence on PUMA: green—increase in its intracellular level; red—decrease in its level or inhibition of its function. Abbreviations: EBV—Epstein–Barr virus; HSV-1—herpes simplex virus type 1; HBV—hepatitis B virus; HIV-1—human immunodeficiency virus type 1; MeV—measles virus; ZIKV—Zika virus; DENV—dengue virus; PUMA—p53-upregulated modulator of apoptosis; Bax—Bcl-2-associated X protein; Bak—Bcl-2 homologous antagonist/killer; Apaf-1—apoptotic protease-activating factor 1; mTOR—mechanistic target of rapamycin; Cdk1—cyclin-dependent kinase 1; FOXO3a—forkhead box O3a; BHRF1—viral Bcl-2 homolog encoded by EBV; miR-BART5—microRNA BamHI A rightward transcript; ER—endoplasmic reticulum; UPR—unfolded protein response; NS5—non-structural protein 5; NF-κB—nuclear factor κB; Env—envelope glycoprotein; CD4—cluster of differentiation 4; gp120—glycoprotein 120; HCC—hepatocellular carcinoma; HBx—HBV X protein.

Viral infections may exert either pro-apoptotic or pro-survival effects on the cell [71,72,73,74,75,76,77,78,79,80,81,82,83,84,85,86,87,88,89,90,91,92,93,94,95,96,97,98,99]. Consequently, virus–host interactions can markedly elevate intracellular PUMA levels or, conversely, diminish the expression of its gene and/or inhibit the activity of the protein [98,100,101,102,103,104,105]. Figure 1 illustrates how distinct viral pathogen–Epstein–Barr virus (EBV), Herpes simplex virus type 1 (HSV-1), Hepatitis B virus (HBV), Human immunodeficiency virus type 1 (HIV-1), Measles virus (MeV), ZIKV, and Dengue virus (DENV)–affect PUMA, while Table 1 summarizes the corresponding data.

### 4.1. PUMA in EBV Infections

EBV, a representative of the *Orthoherpesviridae* family [106], is an infectious agent highly prevalent among humans. The majority of people have been exposed to this pathogen, resulting in a high percentage of carriers—up to 95% [107]. The virus may infect epithelial cells as well as immune cells, such as T and B lymphocytes and natural killer (NK) cells. Different expression patterns of several viral products, including proteins and non-coding RNAs, enable EBV to establish distinct latency programs in infected cells. These phenomena are responsible for long-term pathogen persistence and often contribute to oncogenesis, leading to the development of malignant diseases such as Burkitt’s lymphoma (BL), Hodgkin’s lymphoma T-cell lymphoma, T/NK-cell lymphoma, nasopharyngeal carcinoma (NPC), or gastric carcinoma (GC). The genome of EBV is a linear double-stranded DNA (dsDNA) molecule, containing 90 open reading frames (ORFs), and housed within an icosahedral nucleocapsid. This structure is separated from the external environment by a tegument and a lipid envelope, the latter studded with glycoproteins [100].

EBV may affect host cell viability through a diverse set of factors, including both viral proteins and non-coding RNAs. The first category of pathogen-derived products includes the BamHI fragment H rightward-facing (BHRF1) molecule. Meanwhile, the non-coding RNAs comprise EBV-encoded small RNAs (EBERs) and microRNAs (miRNAs), such as BamHI A rightward transcripts (miR-BARTs) and miR-BHRF1s. Both BHRF1 and miR-BART5 have been found to counteract apoptosis by targeting PUMA [100].

Fitzsimmons et al. [101] delved into the molecular mechanisms responsible for the survival of EBV-infected BL cells, a phenomenon that is a key process in cancer pathogenesis. The team compared BL clones carrying the pathogen with those that had lost the virus. The BL cells were subjected to 48 h stimulation with ionomycin to trigger the intrinsic, mitochondria-dependent apoptotic pathway, and treated with the pan-caspase inhibitor Q-VD.OPh to block terminal caspase activation, thereby restricting apoptosis to early stages and enabling their detailed analysis. The cells were subsequently analyzed by Western blot, and among the 17 proteins examined, Bcl-2-interacting mediator of cell death (Bim) and PUMA showed significantly higher levels in EBV-loss cells compared to EBV-positive clones. Importantly, silencing of Bim and PUMA by small hairpin RNA (shRNA) in BL cells did not alter apoptosis levels, as this knockdown overlapped with the effect of the viral Latency I products, including EBNA1, EBERs, and BART microRNAs [101].

Choy et al. [108] previously demonstrated that the non-coding RNA miR-BART5 contributes to the downregulation of PUMA. Sequence complementarity analysis, combined with a luciferase reporter assay, identified a miR-BART5 target site within the 3′ untranslated region (3′ UTR) of the *PUMA* gene. Alignment of miR-BART5 and the predicted target sequence in the 3′ UTR revealed perfect complementarity over the 7-nucleotide seed region. Despite five mismatches in the central region of the alignment, the base-pairing in the seed region appeared sufficient to mediate miR-BART5-dependent repression of the target gene. It is worth noting that the complementary sequence in the 3′ UTR of *PUMA* is highly conserved, as demonstrated in primates (chimpanzee and rhesus monkey) and mouse. To further validate the role of miR-BART5 in suppressing PUMA-dependent apoptosis, the team assessed its abundance in EBV-positive C666-1 cells, an NPC-derived epithelial line, alongside the EBV-negative NPC line HK1. Another tested sample was HK1, which became EBV-positive (HK1/EBV) after acquiring the virus from Akata-1 cells during co-incubation. The panel of models was completed with the GC-derived AGS cell line, which, upon EBV infection, gave rise to AGS-BX1. All EBV-positive systems, namely C666-1, HK1/EBV, and AGS-BX1, were found to produce miR-BART5, in contrast to EBV-negative HK1. These findings link elevated miR-BART5 levels to NPC and GC phenotypes [108].

Follow-up experiments were aimed at assessing the influence of miR-BART5 on PUMA. EBV presence and expression of miR-BART5 correlated with reduced PUMA levels. In HK1/EBV cells, the abundance of PUMA—specifically the α and β apoptotic isoforms—was decreased by approximately 2–2.5-fold compared with HK1. PUMA β levels were markedly diminished in C666-1 cells in relation to NP69, a non-tumorigenic epithelial line. Treatment with an anti-miR-BART5 oligonucleotide in C666-1 and AGS-BX1 cells led to a marked increase in PUMA. Conversely, transfection of EBV-negative cell lines HeLa and HK1 with pre-miR-BART5 downregulated the β-isoform of this protein. The research was not limited to cell lines; instead, it also included tissue samples collected from patients with NPC. Several of these samples exhibited moderately or severely reduced PUMA levels compared with non-tumorigenic tissues. Previous analyses also reported elevated miR-BART5 levels in NPC samples. Together, these observations suggest a possible association between miR-BART5 presence and diminished PUMA levels in undifferentiated NPC tissues, consistent with the functional effects observed in cell line models [108].

Furthermore, reduced PUMA levels in C666-1, AGS/BX1, and HK1/EBV cells modulated their response to doxorubicin treatment relative to the corresponding EBV-negative lines (NP69, AGS, and HK1, respectively). The deficiency of this pro-apoptotic protein markedly decreased cleavage of poly(ADP-ribose) polymerase (PARP), a molecular hallmark of apoptosis. Terminal deoxynucleotidyl transferase dUTP nick end labeling (TUNEL) assay revealed that inhibition of miR-BART5 induced apoptosis in C666-1 cells [108].

Fitzsimmons et al. [101] also demonstrated the influence of miR-BART5 on PUMA levels in human embryonic kidney 293 (HEK293) cells. Similarly to the effects observed by Choy et al. [108] in NPC and GC lines, transfection of HEK293 cells with a lentiviral vector encoding miR-BART5 decreased PUMA levels, as shown by immunoblotting. However, the effect of miR-BART5 on this apoptotic protein is context-dependent: in EBV-loss BL clones, miR-BART5 alone did not alter PUMA abundance. Only reinfection with EBV resulted in decreased PUMA levels, indicating a cooperative effect of distinct viral products, including EBNA1, EBERs, and miR-BARTs, impending the apoptosis activation [101].

The follow-up study by Fitzsimmons et al. [102] identified PUMA, together with other pro-apoptotic Bcl-2 family members (Bim, BH3 interacting domain death agonist (Bid), and Bak), as targets of the BHRF1 protein. This conclusion was supported by experiments in human BL and murine Eµ-Myc lymphoma-derived cell lines, both representing c-Myc-dependent lymphomas, with BL as an example. Wp-restricted BL cells, characterized by BHRF1 expression, showed increased resistance to apoptotic stimuli (ionomycin or anti-IgM) compared with Latency I BL cells, which lack BHRF1. The combination of site-specific mutagenesis and gene knockout approaches allowed identification of amino acid residues in BHRF1 essential for binding its targets. Phenylalanine residue at position 72 (Phe72) was shown to be critical for interaction with PUMA. These results indicate that in c-Myc-driven aggressive lymphomas, BHRF1 protects cells through synergistic suppression of multiple pro-apoptotic proteins [102].

Taken together, research to date shows that targeting PUMA affects cell viability in various cellular, tissue, and pharmacological contexts and contributes to viral persistence as well as to the pathogenesis of EBV-associated diseases. The pathogen may interfere with PUMA at multiple levels—by repressing its gene transcription or, post-translationally, by inhibiting the protein through direct sequestration by a viral anti-apoptotic factor.

### 4.2. PUMA in HSV-1 Infections

HSV-1, a pathogen belonging to the *Orthoherpesviridae* family [109], is characterized by its ability to establish persistent infections in host cells, particularly neurons. The latent state of the virus facilitates its spread within the population. Epidemiological studies indicate that, in 2016, asymptomatic infections were present in 67% of the global population below 50 years of age, corresponding to approximately 3.7 billion individuals [110,111,112]. The host immune system usually keeps the pathogen under control, preventing clinical manifestations or restricting them to mild, non-life-threatening symptoms [110]. However, in some cases, the infection can lead to the development of severe diseases, including herpes simplex encephalitis (HSE), herpes simplex keratitis and neonatal herpes [110,112]. HSV-1 is a DNA virus, with the virion composed of linear dsDNA, icosahedral capsid, tegument, and lipid envelope decorated with glycoproteins [113].

Despite its tendency to establish latency, HSV-1 is also capable of inducing apoptosis. The dual nature of HSV-1 infection, encompassing both latent and lytic replication cycles, underlies a wide spectrum of clinical manifestations, with the most severe outcomes linked to the pro-apoptotic activity of the virus. Several viral products with anti-apoptotic functions have been identified, including infected cell proteins (ICP)4, 22, and 27, the latency-associated transcript (LAT), the unique short (US)3 kinase, as well as glycoproteins gD and gJ. HSV-1 can prevent host cell death via nuclear factor κB (NF-κB)-dependent signaling, which stimulates the production of a set of prosurvival molecules such as FLIP, cellular inhibitor of apoptosis protein 2, and survivin. The viability of HSV-infected cells is further supported by anti-apoptotic members of the Bcl-2 family [114]. However, in certain cases, viral infection can culminate in apoptosis of immune system cells, including monocytes, T lymphocytes, and dendritic cells [114,115,116,117,118,119]. Additionally, neuronal cell death has been observed following HSV-1 infection, both in rat hippocampal cultures and in brain tissues from patients diagnosed with HSE. Given its contribution to the pathogenesis of HSV-induced neural disorders, the mechanisms underlying HSV-dependent neuronal apoptosis warrants further investigation [119].

Pappaianni et al. [114] studied the molecular events underlying mitochondria-dependent apoptosis in HSV-1-infected cells, including monocytes, fibroblasts, and colon carcinoma-derived cells. Their research identified PUMA as the key protein mediating the switch of intracellular signaling toward Bax/Bak-driven MOMP. Firstly, the team used human U937 monocytes to examine the role of Bax and Bak in virus-mediated apoptosis under NF-κB pathway suppression. After transfection with a vector encoding the NF-κB inhibitor IκBα and infection with HSV-1, Bax- and/or Bak-deficient cells exhibited resistance to apoptosis, whereas their counterparts producing these proteins succumbed to PCD I. These findings demonstrated that viral infection, in the absence of NF-κB-dependent protection, can trigger the cascade of molecular events involving Bcl-2 family apoptotic regulators, ultimately leading to MOMP. The role of Bax and Bak in HSV-1-driven PCD I was further confirmed in a different experimental model. Mouse embryonic fibroblasts (MEFs) immortalized via SV40 T antigen (TAg) underwent apoptosis following viral infection, whereas knockout of the *Bax* and *Bak* rendered the cells insensitive to HSV-1-mediated apoptotic stimulation. The necessity of Bax and Bak for HSV-1-induced PCD I was also demonstrated in mouse factor-dependent monocytes (FDMs) and the human colon cancer cell line HCT116. These findings prompted a systematic search for the molecular sensor responsible for initiating the signaling cascade that culminates in the death of infected cells. Different gene knockouts in TAg+ MEFs, as well as in 3T9 MEFs, allowed the exclusion of a set of candidate BH3-only proteins, including Bid, Bcl-associated death promoter (Bad), Bik, Bim, and Noxa. In contrast, deletion of *PUMA* conferred resistance to apoptosis in 3T9 MEFs, FDMs, and HCT116, highlighting its critical role as the molecular mediator of HSV-1-driven, mitochondria-dependent PCD I. Similarly, partial silencing of PUMA with shRNA conferred protection in both 3T9 and TAg+ MEFs. Further analyses in MEF models allowed reconstruction of an intriguing sequence of molecular events in the trail of HSV-1 infection, with up-regulation of *PUMA* gene transcription following, but not preceding MOMP. The increase in *PUMA* mRNA was determined by the accessibility of Bax and Bak—cells lacking these proteins did not exhibit altered levels of *PUMA* transcript. Interestingly, the amount of PUMA protein continued to rise as infection progressed, even in the absence of Bax and Bak. It suggests that the pathogen may promote apoptosis by affecting PUMA at posttrancriptional level, while transcriptional upregulation occurs downstream of MOMP and is dispensable for initiating cell death. Interestingly, knockout of the genes encoding p53, p73, and p65 NFκB excluded the involvement of these proteins in HSV-1-induced apoptosis. This raised the question of the exact mechanism underlying PUMA upregulation and highlighted the need for a precise reconstruction of the intracellular signaling events leading to the death of HSV-1-infected cells [114].

PUMA has also been shown to contribute to the resolution of the immune response following effective clearance of HSV-1. In this context, antigen-specific T lymphocytes were found to undergo apoptosis via the mitochondria-dependent pathway, as part of a phenomenon aligning the host defense with the organism’s needs. Fischer et al. [120] used mouse mutants lacking genes encoding various members of the Bcl-2 family, including PUMA, Bim, Noxa, Bid, and Bad, to identify the factors responsible for the depletion of CD8^+^ T cells. After 2–4 weeks of HSV-1 infection, the absence of either PUMA or Bim affected the number of these lymphocytes in the spleen, which remained substantially higher than in wild-type animals. These results were corroborated by in vitro experiments. CD8^+^ T lymphocytes isolated from the mouse spleen, under conditions of viral infection and cytokine deprivation, displayed lower levels of apoptosis when lacking PUMA or Bim. This finding suggests an association between the reduction in prosurvival cytokines, the pro-apoptotic activities of these Bcl-2 family members, and the depletion of the aforementioned immune cells during the resolution phase of the antiviral response [120].

### 4.3. PUMA in HBV Infections

HBV is a small, enveloped DNA virus that belongs to the *Hepadnaviridae* [121]. Worldwide, HBV infection impacts more than 250 million individuals and results in over one million fatalities annually due to progressive liver disease and hepatocellular carcinoma (HCC) [122]. The pathogen may spread through contact with infected blood or bodily fluids. In regions of high endemicity, vertical transmission from mother to child represents the predominant route of infection, whereas horizontal transmission in adulthood occurs less frequently. While most infections in adults resolve independently, infections acquired at birth or in early childhood frequently lead to chronic conditions [121]. HBV genome consists of a partially double-stranded, relaxed circular DNA (rcDNA) that is approximately 3.2 kb long and encodes four overlapping ORFs: S, C, P, and X, which encode the viral surface protein (HBs), the core protein (HBc) and the secreted e antigen (HBe), the polymerase, and the X protein (HBx). The infectious virion, known as the Dane particle, consists of an icosahedral nucleocapsid wrapped in a lipid envelope that holds the hepatitis B surface antigen (HBsAg). HBV produces large quantities of non-infectious subviral particles composed solely of HBsAg, serving as decoys that modulate immune recognition [122].

HBV specifically targets the liver, infecting hepatocytes through the interaction between the preS1 domain of its large HBs (L-HBs) and the host receptor sodium taurocholate cotransporting polypeptide (NTCP) [121]. The initial viral attachment occurs through low-affinity binding to heparan sulfate proteoglycans, such as glypican-5, which enables a subsequent high-affinity interaction with NTCP [122]. Upon receptor-mediated endocytosis, the nucleocapsid is released into the cytoplasm and transported to the nucleus, where the rcDNA is repaired to form covalently closed circular DNA (cccDNA). This episomal minichromosome serves as the transcriptional template for all viral RNAs and ensures long-term persistence within infected hepatocytes [121].

During chronic infection, HBV DNA can integrate into the host genome (iDNA), along with cccDNA. Although iDNA cannot support complete replication, it retains the Sp1, Sp2, and X promoters, which drive the expression of HBs and HBx. These integration events create chimeric viral-host transcripts that can modify host gene expression and contribute to oncogenic transformation. Therefore, persistent transcription from iDNA serves as a reservoir for viral antigens and represents a considerable obstacle to achieving a functional cure [122].

Clinically, HBV infection ranges from acute, self-limiting hepatitis to chronic liver disease and HCC [121,123,124,125,126,127,128,129,130,131,132,133,134,135,136,137]. Fulminant hepatitis occurs in about 1–2% of acute infections, primarily driven by an excessive immune response that causes hepatocyte apoptosis and leads to multiorgan failure [138]. Chronic hepatitis B is characterized by continuous HBsAg expression and can progress to fibrosis, cirrhosis, and ultimately HCC. Various mechanisms facilitate the development of HCC in individuals infected with HBV, including ongoing inflammation, oxidative stress, and the integration of viral DNA into the host genome. This integration disrupts host gene regulation and leads to genomic instability [121]. Among the viral proteins, HBx functions as a multifunctional regulator that modifies host signaling pathways, interferes with DNA repair, and reprograms apoptotic responses [139].

HBV is generally considered a non-cytopathic virus, with hepatocellular damage mainly resulting from immune-mediated apoptosis and the cytotoxic effects of viral proteins [121]. The multifunctional HBx protein serves as the principal viral regulator of apoptosis, exhibiting both anti-apoptotic and pro-apoptotic effects depending on its level and the cellular context [139]. The pro-survival properties of HBx may be linked to its potential influence on PUMA. HBx can directly bind to and inactivate p53, and—since p53 can promote PUMA expression—this inactivation is likely to reduce transcription of the gene, thereby suppressing apoptosis during the early stages of infection [103,138,140]. Moreover, at low expression levels, HBx activates NF-κB signaling, leading to the upregulation of anti-apoptotic proteins, including A20 and Bcl-xL, which are direct inhibitors of p53 and promote cell survival and viral persistence [139,141]. In contrast, high levels of HBx expression suppress NF-κB activity and enhance p53-dependent apoptosis, ultimately leading to mitochondrial dysfunction and cytochrome c release [139].

As mentioned earlier, HBV infection may lead to the development of virus-associated liver malignancy, HCC. Pro-apoptotic proteins generally perform anti-oncogenic functions by promoting the death of transformed cells; however, the role of PUMA in HCC pathogenesis appears ambiguous. Ahn et al. [109] reported increased PUMA levels in HBV/hepatitis C virus (HCV)-associated HCC tissues (mainly HBV) in 10 of 20 patients, compared with non-tumor tissues from the same individuals, whereas in the remaining cases, PUMA levels did not differ between matched samples. According to the authors, these results may suggest that PUMA contributes to HCC progression, possibly by exerting survival pressure on transformed cells, thereby favoring the selection of the ones with the greatest resistance [104].

Peng et al. [103] performed a comparative analysis of human HCC and paired non-tumor tissue samples to examine the synthesis and intracellular distribution of the PUMA protein by immunohistochemistry, with the integrated optical density (IOD) used as a quantitative measure. The study encompassed 80 HBV-positive patients, 26 of whom exhibited recurrence-related factors, such as bile duct tumor thrombus (BDTT), portal vein tumor thrombus (PVTT), and multiple satellite lesions. The sample composed in this way enabled the researchers to determine the association between *PUMA* expression levels and the risk of disease relapse. Contrary to Ahn et al. [104], this study showed either the increase in levels of PUMA in transformed hepatocytes, or the drop of this protein, dependent on the case, highlighting the heterogeneity of *PUMA* expression across studies. Elevated PUMA levels in HCC tissues (compared with non-tumor samples from the same individuals) were linked to lower tumor-node-metastasis (TNM) stages, and were also associated with the absence of factors related to disease recurrence. Moreover, further analyses suggested that PUMA influenced the prognosis of HBV-positive HCC patients, as its elevated levels in tumor tissues were correlated with disease-free survival (DFS) and overall survival (OS). The association between PUMA levels in HCC tissues and disease outcomes and the molecular mechanisms regulating *PUMA* expression await further investigation [103].

### 4.4. PUMA in HIV-1 Infections

HIV-1, a member of the *Retroviridae* family [142], is the causative agent of acquired immunodeficiency syndrome (AIDS) [143,144,145]. AIDS remains a significant global public health concern, having caused approximately 44.1 million deaths to date. The transmission of HIV-1, resulting from exposure to infected blood or genital secretions, or from an infected mother to her child during pregnancy, labor, delivery, or breastfeeding, continues to occur in every country around the world. It was estimated that by the end of 2024, 40.8 million individuals were infected, with 65% of this population residing in the WHO African Region. In 2024, roughly 630,000 people died from HIV-related causes, while an estimated 1.3 million individuals were newly diagnosed with the virus [146].

The HIV-1 genome consists of two copies of a single-stranded, positive-sense RNA (ssRNA+) molecule, each about 9.8 to 10.0 kilobases in length. Within the virus particle, the RNA is protected by a conical capsid that houses essential viral enzymes, including reverse transcriptase and integrase. The structure of the virus features a lipid envelope that is derived from the host cell, along with viral proteins and the RNA genome. This host-derived lipid envelope is crucial for the assembly and function of the virus, as it incorporates viral glycoproteins and forms the outer layer of the virus particle [147,148,149].

The hallmark of AIDS progression is the progressive depletion of CD4^+^ T lymphocytes, which weakens the immune system and predisposes patients to immunodeficiencies, autoimmune phenomena, and tumors [150,151,152,153,154,155]. HIV-1 infection is characterized by the activity of envelope glycoproteins (gp120/gp41, envelope glycoprotein complex; Env), which mediate viral entry through interactions with CD4 and chemokine receptors, typically CCR5 and CXCR4 [156,157]. In addition to productive infection, HIV establishes long-lived latent reservoirs in memory CD4^+^ T cells. These reservoirs pose a significant challenge to eradicating the virus [158]. Additionally, HIV-1 can also infect dendritic cells, serving as a vector to transmit the virus to memory CD4^+^ T cells [159].

Depending on the tropism, HIV strains can be classified as either non-syncytium-inducing (NSI; CCR5-tropic) or syncytium-inducing (SI; CXCR4-tropic) [160,161]. The shift from NSI to SI viruses is associated with advanced disease progression and poor prognosis, as SI variants exhibit enhanced cytopathic activity and readily induce the formation of multinucleated cells (syncytia) [162,163]. While syncytia are frequently observed in vitro in peripheral blood mononuclear cell (PCBM) cultures and T-cell lines, their detection in vivo has mainly been limited to the central nervous system (CNS). Infected microglia and macrophages form multinucleated giant cells, a neuropathological hallmark of HIV-associated dementia [163,164,165].

The cytopathic effect of syncytia is closely linked to activation of apoptosis. Env-CD4 interactions trigger a sequence of intracellular events involving NF-κB activation, mechanistic target of rapamycin (mTOR) signaling, and phosphorylation of p53 at serine 15 and serine 46. This results in aberrant cell cycle entry mediated by cyclin B1 and activation of mitochondrial pathways of PCD I. Although NF-κB is classically regarded as an anti-apoptotic transcription factor [166,167], in this context it enables mitotic progression and acts upstream of p53 activation. Additional viral proteins, including Tat, Rev, Nef, Vif, Vpr and Vpu, may cooperate with Env in stimulating pro-apoptotic cascades [168,169,170].

Central to this process is the activation of PUMA, as both transcriptional profiling and functional studies identified PUMA as one of the principal effectors of HIV-1-induced apoptosis [171]. Env-driven syncytia exhibit a defined signaling cascade, in which NF-κB activation and phosphorylation of its inhibitor IκB (Ser32/36) precede aberrant cyclin B1/cyclin-dependent kinase 1 (Cdk1) activity and the sequential phosphorylation of p53 on Ser15 and Ser46. This ordered activation, confirmed in both in vitro syncytia and lymph node biopsies from HIV-1 carriers, culminates in the robust transcription of *PUMA* driven by p53. Transcriptional profiling using microarray and macroarray approaches consistently identified PUMA, together with Bax and PIG8, as among the most strongly upregulated p53 target genes during syncytia formation, with a ≥2-fold induction validated by quantitative RT-PCR and immunoblotting [171].

Functionally, PUMA induction correlates with conformational activation of Bax and Bak, MOMP, cytochrome c release, and caspase activation. Antisense anti-*PUMA* oligonucleotides abolish Bax/Bak activation, prevent cytochrome c release, and block chromatin condensation, underscoring its essential role in the execution of apoptosis [171]. RNA interference experiments revealed a functional hierarchy between Bak and Bax, in which Bak activation is required for Bax conformational change, but not vice versa, positioning PUMA as the upstream trigger of this cascade. While p53-dependent transcription is a major driver of *PUMA* expression, alternative p53-independent pathways also contribute. For example, gp120 variants induced *PUMA* expression and apoptosis in p53-deficient U937 cells, and transfection with wild-type p53 accelerated gp120-mediated *PUMA* induction, indicating that p53-dependent and -independent routes can cooperate to amplify apoptosis [171].

Clinical studies further highlight the importance of PUMA in HIV-1 infection. Elevated PUMA expression was observed in lymph nodes and circulating CD4^+^ T lymphocytes from untreated HIV-1-infected patients, with particularly high expression in CD4^+^ T cells and CD14^+^ monocytes [171]. Importantly, PUMA levels showed a positive correlation with viral titers, with the strongest induction observed in patients with high viral loads (>20,000 copies/mL). Immunoblotting confirmed elevated PUMA protein in CD4^+^ cells, while longitudinal analyses demonstrated that antiretroviral therapy restored PUMA expression to baseline levels. This decline was paralleled by a recovery of CD4^+^ T-cell homeostasis and a reduction in the spontaneous apoptosis of circulating lymphocytes. Collectively, these findings establish PUMA as a central mediator of CD4^+^ T-cell depletion in AIDS, suggesting its potential as a biomarker for disease progression and therapeutic efficacy [171].

### 4.5. PUMA in MeV Infections

MeV is a member of the *Paramyxoviridae* family [172]. Although an effective vaccine for the prevention of infection has been licensed for decades, it has not stopped the spread of the pathogen, and measles, a MeV-driven disease, remains a global health problem [173]. The limited coverage of vaccination enables the virus to disseminate, infecting approximately 9,000,000 people worldwide in 2022, mainly children under 5 years old, with an exceptionally high mortality: the number of reported deaths exceeded 135,000 [174]. The effective spread of MeV occurs via the respiratory tract, through both droplet and airborne transmission. In the host lungs, MeV infects immune cells, including dendritic cells, which act as vehicles to transport the virus to the local lymph nodes and subsequently to its secondary targets, the T lymphocytes. Next, MeV replicates in the host immune cells and undergoes further dissemination, colonizing various epithelial and submucosal tissues, including those of the oral cavity, upper respiratory tract, and skin [175]. MeV is an RNA virus, with the virion consisting of nonsegmented negative-sense ssRNA (ssRNA-) arranged with nucleoprotein (N) into a helical ribonucleocapsid that is associated with phosphoprotein (P) and the viral polymerase (L), and surrounded by matrix protein and a lipid envelope adorned with glycoproteins [85,174,176].

MeV has been shown to possess both pro-apoptotic and anti-apoptotic activities, depending on the infected cell type and stage of infection. It induces apoptosis in immune cells, particularly PBMCs, in a caspase-dependent manner. Its ability to induce cell death makes it a promising candidate for use as an oncolytic agent in anticancer therapy. The activity of the N protein has been linked to both oxidative stress and caspase-3 activation during MeV infection, implicating that it contributes to the pro-apoptotic properties of the virus. On the other hand, the viral P protein displays the opposite activity. It has been found to increase the transcription of two anti-apoptotic cellular genes in HeLa cells, resulting in a substantial rise in mRNA levels encoding both Bcl-2 and Bcl-xL proteins, and a decrease in the percentage of apoptotic cells [85].

Moreover, another pro-survival mechanism employed by MeV to manipulate host cell viability has been linked to changes in PUMA level. Cruz et al. [177] investigated the V protein-dependent mechanisms by which MeV disrupts the host’s antiviral response pathways. Co-precipitation assays revealed the affinity of the viral V protein to two members of the p53 family. The MeV V protein was found to bind to the DNA-binding domains (DBDs) of the p53 and p73 factors. These findings prompted the authors to hypothesize that the V protein may influence the viability of infected cells. To verify this assumption, they used the 2fTGH fibrosarcoma cell line transduced with lentiviral vectors engineered to express the MeV V protein. Treatment with doxycycline was employed to induce apoptosis. As expected, the presence of the V protein counteracted this apoptotic stimulus, substantially reducing the percentage of apoptotic cells, as measured by propidium iodide staining and flow cytometric analysis of fragmented DNA, and confirmed by PARP cleavage. Further investigations revealed significant changes in the transcription of two genes, each encoding a cellular pro-apoptotic factor, in the presence of ectopically produced MeV protein, compared to non-transduced controls. Both *Bax* and *PUMA* mRNAs were significantly reduced in MeV V-positive cells, with *PUMA* transcript levels decreasing by more than twofold. Western blot analysis of PUMA protein levels in doxycycline-treated human 293T cells demonstrated that the presence of MeV V is associated with reduced PUMA synthesis. Assessment of p53- and p73-dependent luciferase reporter activity indicated that MeV V protein selectively inhibits p73-mediated transcription, while p53 activity remains unaffected. The V protein-deficient mutant ultimately displayed markedly greater cytopathicity toward 2fTGH and Vero cells than the full-genome strain, suggesting a link between virus-induced cytopathic effects and the viability of infected cells, which was associated with PUMA levels. The above-mentioned results suggest that counteracting apoptosis may be part of the pathogen’s strategy for efficient replication. By rescuing cells from p73/PUMA-mediated PCD I, MeV may preserve the natural environment required for its replicative cycle [177]. According to the scientific literature, the V protein may interfere with the host antiviral response in paramyxoviruses, as demonstrated not only for MeV but also for simian virus 5 (SV5). By disrupting interferon (IFN) signaling and promoting cell survival, the V protein fosters the development of infection. It would be worth investigating whether PUMA levels are altered during SV5 infection. The influence of MeV on PUMA levels in different cellular contexts and at distinct stages of systemic infection also warrants thorough examination [177,178,179].

### 4.6. PUMA in IAV Infections

Influenza A virus (IAV), a pathogen belonging to the *Orthomyxoviridae* family [180], is capable of infecting a broad spectrum of hosts, including dogs, horses, pigs, seals, minks, ferrets, bats, and birds. Apart from animals, it also poses a threat to humans as a widespread agent of seasonal respiratory infections, which are difficult to prevent due to the extremely high genetic variability of the virus [17]. The manifestations of IAV-induced disease may be limited to benign symptoms such as cough and fever, however, more severe outcomes can also occur, including acute respiratory distress syndrome (ARDS) and neurological complications such as encephalitis and encephalopathy [17,99,181,182]. The genetic heterogeneity of strains within the IAV species results from the segmentation of its genome, a feature that facilitates RNA reassortment and the emergence of new viral variants, thereby necessitating systematic annual vaccination to maintain immune preparedness [183,184,185]. The ssRNA- genome of IAV is contained within an icosahedral capsid and surrounded by an envelope studded with two glycoproteins, hemagglutinin and neuraminidase, the combination of which determines the strain identity [17].

The progression of pathogen-host interactions at both cellular and systemic levels has been associated with the survival of infected mast cells (mastocytes) [105]. They are important components of the innate arm of the immune system, with the ability to modulate the activation of the adaptive branch by influencing T-lymphocyte stimulation [186]. Mastocytes produce a variety of immunomodulatory compounds and accumulate them in specialized intracellular granules. These substances include proteases [187,188,189,190,191,192,193,194], proteoglycans [191], and bioactive amines, the latter represented primarily by histamine [195,196,197,198,199,200,201]. By releasing this mediator, mast cells may contribute to the development of asthma. Mastocytes are well known to participate in allergic reactions and autoimmune-driven inflammatory responses [186,202]. On the other hand, they may exert a favorable impact on host condition, participating in processes such as detoxification, neuroprotection, tissue renewal, and, importantly, recruitment and/or activation of immune cells. By engaging these immune components or acting directly, mastocytes can achieve a potent antimicrobial effect. Cytokine-driven recruitment of NK cells, natural killer T (NKT) cells, and cytotoxic T (Tc) cells, together with the secretion of lipid-envelope-disrupting cathelicidins, constitute mast cell-mediated mechanisms directed at viral pathogen elimination [186]. However, mastocyte-induced inflammation may also promote viral pathogenesis. In the course of IAV infection, mast cells can intensively release histamine, tryptase, and a set of immunoregulatory proteins, including interleukin (IL)-6, IL-18, TNF-α, IFN-γ, and monocyte chemoattractant protein-1/C-C motif chemokine ligand 2 (MCP-1/CCL2) [105,203,204]. By mediating a strong inflammatory response through their secretory activity, mastocytes shape the pathogenesis of IAV-driven disease, contributing to hyperinflammation and subsequent lung injury [105,203]. Research by Liu et al. [105] suggests a role for PUMA protein in mast cell apoptosis. The team examined three IAV strains, each representing a distinct viral subtype: H1N1 (A/WSN/33), H5N1 (A/Chicken/Henan/1/04), and H7N2 (A/Chicken/Hebei/2/02). P815, a mastocytoma cell line originating from a mouse, underwent apoptosis in response to each of the above-mentioned pathogens. Noticeably, the levels of the pro-apoptotic Bcl-2 family proteins PUMA and Bim were consistently increased in IAV-infected cells, as demonstrated by Western blot analysis. This result was in keeping with the observed caspase-9 cleavage, indicating the intrinsic apoptotic pathway as the predominant mechanism of mastocyte death. Further analyses revealed the role of PCD I in promoting IAV replication and virus-driven inflammation. Exposure of IAV-infected P815 cells to a pan-caspase inhibitor (Z-VAD-fmk) or a caspase-9 inhibitor (Z-LEHD-fmk) diminished viral titers and reduced the secretion of four pro-inflammatory cytokines: IL-6, IL-18, TNF-α, and MCP-1/CCL2 [105].

In general, programmed cell death during viral infection may act as a double-edged sword, either impairing or promoting viral spread. IAV exemplifies the latter scenario, exploiting apoptosis to facilitate propagation. The rapid kinetics of IAV replication allow the production of progeny virions to occur before apoptosis is completed, thus rendering the virus largely independent of prolonging host cell viability. Moreover, apoptotic signaling in mastocytes is tightly coupled to their pro-inflammatory response during IAV infection, indicating a potential role for PUMA in amplifying cytokine release and the resulting pathology [105].

### 4.7. PUMA in Flaviviral Infections

Flaviviruses are enveloped ssRNA+ viruses of the *Flaviviridae* family, which includes major human pathogens such as DENV and ZIKV. Their genome, approximately 10.7–11 kb in length, encodes a single open reading frame flanked by 5′ and 3′ untranslated regions. They produce three structural proteins: capsid (C), premembrane/membrane (prM/M), and envelope (E), and seven non-structural proteins (NS1–NS5) [205,206].

DENV circulates as four antigenically distinct serotypes (DENV-1 to DENV-4) and causes about 390 million infections annually, of which 100 million are symptomatic [207]. Clinical manifestations can range from mild dengue fever to severe cases like dengue hemorrhagic fever and dengue shock syndrome. ZIKV, first identified in 1947, became a major global threat after the outbreaks in the Americas during 2015–2016, which were associated with congenital Zika syndrome and Guillain–Barré syndrome [205]. While approximately 80% of infections remain asymptomatic, severe neurological and developmental outcomes are characteristic of prenatal infection [206].

Both DENV and ZIKV exhibit broad cellular tropism. DENV targets hepatocytes, dendritic cells, endothelial cells, and monocytes, with the liver being a crucial site for viral replication and disease impact [207]. In hepatocytes, infection triggers ER stress, activates the unfolded protein response (UPR), and leads to apoptosis, as seen in fatal dengue cases [207].

ZIKV demonstrates strong neurotropism, targeting neural progenitor cells (NePCs) and astrocytes, and can also infect placental trophoblasts. Interactions between the virus and receptors such as Axl, Tyro3, and TIM1 facilitate viral entry. These interactions promote the endocytosis of the E protein-receptor complex [205]. Once the complex is internalized, the acidic environment within the cell induces conformational changes in the E protein, which allows for fusion and the release of the viral genome [206]. This finding indicates that the virus can establish prolonged latency-like states [205].

DENV infection leads to systemic vascular leakage and liver dysfunction, which is associated with widespread apoptosis and metabolic failure in liver cells. The viral NS proteins, along with the accumulation of misfolded prM and E proteins in the ER, create cellular stress and disrupt cytokine regulation. This, in turn, exacerbates inflammatory damage [207].

In contrast, ZIKV pathogenesis primarily focuses on the damage to neural and reproductive tissues. Infection of developing fetal brain tissue leads to neuronal apoptosis, cortical thinning, and microcephaly [205,208]. In adult models, ZIKV has been detected in testes and ocular tissues, associated with infertility and uveitis [205]. On a molecular level, ZIKV induces pathology through ER stress, mitochondrial dysfunction, and the inhibition of interferon signaling. The non-structural proteins NS1, NS4B, and NS5 primarily mediate these effects [205,206].

Both ZIKV and DENV exploit ER stress pathways and the intrinsic mitochondrial death machinery to regulate host apoptosis. In DENV-infected hepatocytes, the dissociation of glucose-regulated protein 78 (GRP78) from ER sensors inositol-requiring enzyme 1 (IRE1), PKR-like ER kinase (PERK), and activating transcription factor 6 (ATF6) can trigger the activation of the UPR. Thepparit et al. [207] used DENV serotype 2 (DENV-2) to infect HepG2 cells and analyze ER-stress markers induced by the virus. This viral challenge resulted in the splicing of X-box binding protein 1 (XBP1), the phosphorylation of translation initiation factor 2 alpha (eIF2α), and the transcriptional induction of *C/EBP homologous protein* (*CHOP*), *Noxa*, and *PUMA*. Semi-quantitative and real-time RT-PCR confirmed a robust upregulation of all three genes, with *PUMA* transcripts increasing earlier than those of *Noxa*. Further analyses showed a concurrent decrease in mitochondrial membrane potential. As shown by Western blot and enzyme-linked immunosorbent assay (ELISA), DENV-2 exposure led to the activation of caspases-4, -7, -8, and -9, thereby linking ER stress to both the intrinsic and extrinsic apoptotic pathways. The coordinated upregulation of *PUMA* and *Noxa* emphasizes their role as essential BH3-only effectors that connect ER stress to mitochondria-mediated cell death [207].

In neuronal cells, ZIKV triggers a comparable apoptotic response. In a recent study by Krishnamoorthy et al. [209], two recombinant ZIKV strains, MR766 and PRVABC59, were examined for their effects on ER stress markers and the viability of SH-SY5Y neuroblastoma cells differentiated into neuron-like cells. Infection increased the cleavage of PARP, caspase-3, and the levels of pro-apoptotic BH3-only proteins, such as PUMA and Bim, and down-regulated anti-apoptotic Bcl-2 family members. ZIKV activated all three branches of the UPR: PERK, IRE1α, and ATF6. It culminated in the phosphorylation of eIF2α and the expression of gene encoding *CHOP*. Excessive ER stress resulted in mitochondrial permeabilization, the release of cytochrome c, and activation of caspase-9. Pharmacological inhibition of ER stress or supplementation with palmitoleate markedly reduced PUMA level, caspase activation, and viral replication, confirming that PUMA-mediated apoptosis contributes to the cytopathic effects of ZIKV [209].

Similarly, Li et al. [208] identified an additional mechanism by which ZIKV infection promotes *PUMA* expression. Using immunoprecipitation-coupled mass spectrometry, and confirming these findings with immunoprecipitation-based approaches, including co-immunoprecipitation and glutathione S-transferase (GST) pull-down assays, the authors demonstrated that the viral NS5 protein interacts with the cellular factor p53. A deeper insight into the molecular mechanism underlying this interplay revealed that the viral NS5 protein binds the C-terminal region of its target through its methyltransferase domain. The interaction between NS5 and p53 enhanced the stability of the latter molecule. Studies on human neural progenitor cells (hNePCs) demonstrated the apoptotic effect of ZIKV infection and enabled the researchers to explore the molecular basis of ZIKV-induced PCD I, a process that contributes to viral pathogenesis by impairing neurogenesis and promoting neonatal microcephaly. As shown by Western blot analysis, the viral challenge resulted in PARP proteolysis, a hallmark of apoptosis. Further investigation using quantitative RT-PCR (RT-qPCR) demonstrated an increase in the transcription of several p53-dependent genes, including those encoding *Noxa*, *p21*, and *PUMA*. The positive effect of ZIKV on *PUMA* mRNA levels was subsequently confirmed in another cell line, HEK293T. Ectopic presence of NS5, and to a smaller degree, NS2A, increased *PUMA* transcript abundance. Finally, transfection of hNePCs with an NS5-encoding vector produced a comparable outcome [208].

Collectively, these findings identify PUMA as a central mediator of apoptosis in both DENV and ZIKV infections, integrating ER stress, UPR activation, and mitochondrial dysfunction into a unified cell-death pathway. In ZIKV-infected cells, a complementary p53-dependent mechanism driving *PUMA* transcription has also been demonstrated. The regulation of mitochondria-mediated apoptosis by viral factors and host metabolic signals, therefore, emerges as a promising therapeutic axis for mitigating flavivirus-induced tissue damage.

## 5. PUMA as a Prospective Therapeutic Target and Biomarker in Viral Infections

As a potent pro-apoptotic regulator, PUMA may determine cell viability and, indirectly, influence viral replication and the overall course of pathogenesis. This Bcl-2 family member has been shown to affect the efficiency of IAV propagation as well as host immune responses, including cytokine storms that contribute to IAV-mediated inflammation [105]. By modulating ER stress-dependent and/or p53-mediated intrinsic apoptosis pathways, PUMA may also play a role in the detrimental effects of ZIKV and DENV infections, such as liver damage and neural disorders [207,208,209]. Therefore, targeting PUMA may represent a promising strategy for mitigating these virus-induced pathologies. Inhibiting this protein may alter cell fate and, consequently, the trajectory of the pathological processes, potentially reducing viral propagation, inflammation, and organ-specific disease symptoms. One compound with demonstrated PUMA-targeting potential is Component 8 (CTZ-8). Feng et al. [210] investigated this molecule in the context of radiotherapy-associated side effects. Inactivation of PUMA mitigated apoptosis in human umbilical vein endothelial cells (HUVECs) exposed to ionizing radiation, and these in vitro observations were confirmed in a mouse model. Administration of CTZ-8 increased the post-irradiation survival of the animals. Although CTZ-8 was developed as a radioprotective agent [210], its mechanism of action suggests broader therapeutic potential in conditions where excessive apoptosis contributes to tissue damage, including viral infections and their associated pathologies.

Another apoptosis modulator with emerging therapeutic relevance is palmitoleate, a monounsaturated fatty acid. Krishnamoorthy et al. [209] demonstrated its neuroprotective effect during ZIKV infection and virus-induced ER stress. As mentioned above, treatment of ZIKV-infected neuron-like SH-SY5Y cells with palmitoleate exerted an anti-apoptotic effect, including a substantial reduction in PUMA levels. This phenomenon resulted from the alleviation of ER stress and the consequent attenuation of the PERK–eIF2α–ATF4–CHOP signaling cascade. A 72 h exposure to 200 µM of this fatty acid markedly decreased the amount of PUMA protein, an effect accompanied by a concurrent reduction in PARP cleavage. Finally, shifting intracellular signaling toward a pro-survival outcome reduced the viral titer not only in SH-SY5Y cells but also in fetal cortical neurons isolated from hSTAT2KI mice [209].

Taken together, these findings indicate that both direct and indirect targeting of PUMA may offer a promising therapeutic avenue to protect infected hosts from pathogens, restraining apoptosis-dependent viral replication and mitigating the detrimental consequences of excessive cell death.

A body of research highlights the role of PUMA in HIV-induced apoptosis of host immune cells and provides grounds to consider indirect, p53-dependent PUMA targeting for preventing virus-driven immunosuppression. Pharmacological studies offer significant evidence that upstream signaling events tightly regulate PUMA activation during HIV-1 infection. Inhibiting NF-κB with the peptide SN50 or with an IκB super-repressor prevents cyclin B1 accumulation, karyogamy, phosphorylation of p53, and subsequent PUMA upregulation in Env-driven syncytia. Similarly, blocking Cdk1 with roscovitine or inhibiting mTOR with rapamycin disrupts the sequential phosphorylation of p53 at Ser15 and Ser46 [211], thereby suppressing its transcriptional activity and the downstream induction of PUMA. Most notably, the p53 inhibitor cyclic pifithrin-α abolishes approximately 85% of the transcriptional changes elicited by Env, including the strong upregulation of PUMA, and prevents syncytial apoptosis. These findings demonstrate that the NF-κB–Cdk1/mTOR–p53 signaling axis is indispensable for PUMA expression in HIV-1 infection, and that pharmacological interference at multiple levels of this cascade can effectively block mitochondrial apoptosis of CD4^+^ T cells [171].

Considering PUMA solely as an antiviral target does not exhaust the potential medical applications stemming from our understanding of this protein. It may also be considered a prognostic indicator in HBV-related oncogenesis—specifically, HCC. As mentioned above, elevated PUMA levels have been associated with lower TNM stages, the absence of recurrence-related factors, and improved DFS and OS, all of which point toward a more favorable prognosis. Thus, monitoring PUMA levels appears to be a promising approach for estimating clinical parameters that predict patient outcomes [103].

Although PUMA appears to be a promising direct or indirect target for antiviral therapies, treatment strategies must consider several limitations and potential risks. These include the possibility of excessive apoptosis inhibition in non-infected cells, challenges in precisely modulating PUMA levels or activity in specific tissues or cell types, and potential side effects from long-term interference with cellular signaling pathways, including p53-dependent cascades and ER stress responses.

### Therapeutic Translation: Current Developments

Efforts to translate PUMA modulation into therapies are still at an early stage. Direct PUMA inhibitors, such as CTZ-8, and indirect modulators like palmitoleate, have demonstrated efficacy in preclinical models but have not yet entered clinical trials. Currently, no PUMA-targeting compounds are registered in clinical trials for viral infections, highlighting a critical gap between mechanistic insights and therapeutic translation.

## 6. Conclusions

The viability of host cells is tightly regulated by both extrinsic and intracellular signals that integrate into a vast, densely interwoven network spanning entire tissues, organs, and systems. Control of cell fate subordinates individual cells to the interests of the multicellular organism, enabling proper and directed morphogenesis. Apoptosis is a key process that ensures the maintenance of organismal health. It allows the host to eliminate old, damaged, malfunctioning, or transformed cells for the benefit of the whole body. It also plays an important—though often ambiguous—role in the context of viral infections.

Virus–host cell interactions may modulate the signaling cascades that constitute both the extrinsic and intrinsic pathways of PCD I. The latter arm centers on mitochondrial integrity, the loss of which represents the key event in this process. The fate of the cell depends on the subtle interplay of pro- and anti-apoptotic factors, including members of the Bcl-2 family. Among them is PUMA, an anti-survival factor of particular importance. Its contribution to mitochondria-dependent apoptosis may shape, influence, and determine virus–host interactions, the propagation of the infectious agent, and the course of pathogen-related diseases. Such involvement has been reported for EBV, HSV-1, HBV, HIV-1, MeV, IAV, ZIKV, and DENV. PUMA-supported apoptosis may facilitate certain viral infections and exacerbate their outcomes. It is partially responsible for immunosuppression in the context of HSV-1 and HIV-1 infection, where it contributes to the depletion of CD8^+^ and CD4^+^ lymphocytes, respectively. During IAV challenge, it promotes mastocyte apoptosis as well as inflammation, thereby triggering a cytokine-mediated response. Research has revealed additional effects of PUMA activity that are detrimental for hosts infected with two ssRNA viruses: this pro-apoptotic member of the Bcl-2 family may enhance the replication efficiency of IAV and HIV, as reflected by substantially higher viral titers. PUMA is also detrimental in flaviviral infections: its activity in DENV-positive hepatocytes and ZIKV-infected neural cells can lead to liver damage and neural pathologies, respectively, the latter including serious disorders such as neurodegeneration and microcephaly. The role of PUMA in promoting viral infections makes it a component of pathogens’ strategies to manipulate host intracellular signaling, tailoring it to the viruses’ needs. HSV-1, HIV-1, DENV, and ZIKV have been found to induce the production of this protein, either via p53-dependent or p53-independent pathways.

On the other hand, the high apoptotic potential of the host cell may serve as a circumstance preventing certain viral infections and pathogen-associated diseases, and in this context, PUMA functions as an anti-viral agent. Consequently, some viruses target it and—contrary to the aforementioned infectious agents—diminish its level or activity. MeV encodes the V protein, a product capable of binding p73, a transcriptional activator of the PUMA gene. In this way, the pathogen decreases PUMA levels, which appears to sustain host cell viability and thus preserve the environment for viral replication. Next, the anti-apoptotic abilities of EBV are responsible for the latency (permissiveness) of infection and contribute to the development of EBV-associated diseases. PUMA down-regulation is part of this process: research has shown that two viral products, the BHRF1 protein and the miR-BART5 transcript, suppress PUMA via direct inactivation and transcriptional repression, respectively.

Changes in PUMA levels have also been observed in HBV/HCV (mainly HBV)-related and HBV-associated HCC. Depending on the patient, the disease resulted in elevated, decreased, or unmodified PUMA levels. The role of this protein in HCC development, however, remains equivocal. One report suggests that PUMA may drive HCC progression by imposing survival pressure on transformed cells and favoring the selection of those with the greatest resistance to apoptosis. Meanwhile, another study associates elevated PUMA levels with better survival prognosis, lower TNM stages, and the absence of factors linked to disease recurrence. The influence of HBV alone on PUMA levels has not been determined yet; however, some information exists regarding its ambiguous effect on p53 signaling. HBV’s HBx protein can dysregulate p53 either indirectly, by modulating NF-κB activity, or directly, by binding to and inhibiting the p53 molecule. The ambiguity of this influence suggests a potentially equivocal effect of the virus on PUMA, as reflected in HCC studies.

The indispensable role of PUMA in viral infections has inspired scientists to explore the therapeutic implications of understanding its intracellular signaling. Direct or indirect targeting of PUMA could reshape both the fate of the cell and the course of infection, thereby affecting disease outcomes. Whether the infectious agent upregulates or suppresses PUMA determines the potential antiviral strategy: pathogens inducing PUMA-dependent apoptosis may be countered by inhibiting PUMA or modulating its upstream regulators. Moreover, for viruses that antagonize PUMA, strategies aimed at restoring pro-apoptotic signaling could be explored in the future. Additionally, PUMA may also be considered a potential marker in HBV-associated HCC.

Despite the undeniable significance of PUMA within the apoptotic molecular network in the context of viral infections, several important questions remain unanswered. Current knowledge gaps arise primarily from the lack of comprehensive in vivo data, particularly from clinical studies, as most available evidence is derived from in vitro systems or animal models. The strong context dependency of virus–PUMA interactions also requires further investigation. Mechanisms underlying divergent effects of viral infection on PUMA expression or activity, such as those depending on the stage of infection or the abundance of viral proteins, as observed for HBV, remain incompletely understood. Moreover, the development of safe and selective PUMA modulators represents a major challenge, as compounds such as CTZ-8 and palmitoleate have not yet been translated into clinically approved therapies. Finally, the potential utility of PUMA as a biomarker warrants prospective validation in well-designed clinical studies.

## Figures and Tables

**Table 1:** The influence of viral infections on PUMA.

Virus	Viral Factor(s)	Mechanism	Apoptotic Profile
EBV	miR-BART5	Direct targeting of *PUMA* mRNA (3′ UTR binding)	Anti-apoptotic
	BHRF1	Direct sequestration of PUMA (protein binding)	Anti-apoptotic
HSV-1	Not clearly identified	Indirect promotion of PUMA protein accumulation independent of p53/p73/NF-κB	Pro-apoptotic
HBV	HBx	Direct binding and inactivation of p53, leading to reduced *PUMA* transcription	Anti-apoptotic
		NF-κB signaling activation, leading to inhibition of p53-dependent *PUMA* induction (low HBx levels)	Anti-apoptotic
		NF-κB activity suppression, leading to enhancement of p53-dependent *PUMA* induction (high HBx levels)	Pro-apoptotic
HIV-1	Env	NF-κB-dependent p53-mediated stimulation of PUMA transcription	Pro-apoptotic
		p53-independent *PUMA* induction	Pro-apoptotic
MeV	V protein	Inhibition of p73-mediated *PUMA* transcription	Anti-apoptotic
ZIKV	Not defined	ER stress/UPR activation	Pro-apoptotic
DENV	Not defined	ER stress/UPR activation	Pro-apoptotic
	NS5	Stabilization of p53 and p53-dependent PUMA induction	Pro-apoptotic

Abbreviations: EBV—Epstein–Barr virus; BHRF1—BamHI fragment H rightward-facing 1; HSV-1—Herpes simplex virus type 1; HBV—Hepatitis B virus; HBx—Hepatitis B virus X protein; NF-κB—nuclear factor kappa B; HIV-1—Human immunodeficiency virus type 1; Env—Envelope glycoprotein complex of HIV-1 (gp120/gp41); MeV—Measles virus; ZIKV—Zika virus; DENV—Dengue virus; NS5—non-structural protein 5; PUMA—p53-upregulated modulator of apoptosis; ER—endoplasmic reticulum; UPR—unfolded protein response; 3′ UTR—3′ untranslated region.

## Data Availability

No new data were created or analyzed in this study.

## Abbreviations

The following abbreviations are used in this manuscript:

3′ UTR	3′ untranslated region
AIF	apoptosis-inducing factor
AIDS	acquired immunodeficiency syndrome
ARDS	acute respiratory distress syndrome
Apaf-1	apoptotic protease-activating factor 1
ATF6	activating transcription factor 6
Bak	Bcl-2 homologous antagonist/killer
Bad	Bcl-2-associated death promoter
Bax	Bcl-2-associated X protein
Bcl-2	B-cell lymphoma 2
Bcl-xL	B-cell lymphoma-extra large
BDTT	bile duct tumor thrombus
BH3	Bcl-2 homology 3 domain
BHRF1	BamHI fragment H rightward-facing protein
Bid	BH3 interacting domain death agonist
Bim	Bcl-2-interacting mediator of cell death
BL	Burkitt’s lymphoma
cccDNA	covalently closed circular DNA
Cdk1	cyclin-dependent kinase 1
cFLIP	cellular FLICE-like inhibitory protein
CHOP	C/EBP homologous protein
CNS	central nervous system
CTZ-8	Component 8
DBD	DNA-binding domain
DFS	disease-free survival
DISC	death-inducing signaling complex
DENV	Dengue virus
dsDNA	double-stranded DNA
EBERs	EBV-encoded small RNAs
EBV	Epstein–Barr virus
ELISA	enzyme-linked immunosorbent assay
Env	envelope glycoprotein complex (gp120/gp41)
eIF2α	eukaryotic translation initiation factor 2 alpha
ER	endoplasmic reticulum
FADD	Fas-associated death domain
FDMs	factor-dependent monocytes
FOXO3a	forkhead box O3
GC	gastric carcinoma
GRP78	glucose-regulated protein 78
GST	glutathione S-transferase
HBc	hepatitis B core antigen
HBe	hepatitis B e antigen
HBs	hepatitis B surface protein
HBsAg	hepatitis B surface antigen
HBV	Hepatitis B virus
HBx	hepatitis B x protein
HCC	hepatocellular carcinoma
HCV	Hepatitis C virus
HEK293	human embryonic kidney 293 cells
HIV-1	human immunodeficiency virus type 1
hNePCs	human neural progenitor cells
HSE	herpes simplex encephalitis
HSV-1	Herpes simplex virus type 1
HUVECs	human umbilical vein endothelial cells
IAV	influenza A virus
ICP	infected cell protein
iDNA	integrated viral DNA
IFN	interferon
IL	interleukin
IOD	integrated optical density
IκB	inhibitor of NF-κB
IRE1	inositol-requiring enzyme 1
LAT	latency-associated transcript
L-HBs	large HBs
MCP-1/CCL2	monocyte chemoattractant protein-1/C-C motif chemokine ligand 2
MEFs	mouse embryonic fibroblasts
MeV	Measles virus
miR-BARTs	BamHI A rightward transcripts microRNAs
miRNAs	microRNAs
MLS	mitochondrial localization signal
MOM	mitochondrial outer membrane
MOMP	mitochondrial outer membrane permeabilization
mTOR	mechanistic target of rapamycin
NF-κB	nuclear factor κB
NKT cells	natural killer T cells
NK cells	natural killer cells
NPC	nasopharyngeal carcinoma
NePCs	neural progenitor cells
NTCP	sodium taurocholate cotransporting polypeptide
NS	non-structural protein
NSI	non-syncytium-inducing
ORFs	open reading frames
OS	overall survival
PARP	poly(ADP-ribose) polymerase
PCBM	peripheral blood mononuclear cell
PCD I	programmed cell death type I
PERK	PKR-like ER kinase
prM/M	premembrane/membrane protein
PUMA	p53-upregulated modulator of apoptosis
PVTT	portal vein tumor thrombus
rcDNA	relaxed circular DNA
ROS	reactive oxygen species
RT-PCR	reverse transcription polymerase chain reaction
RT-qPCR	quantitative RT PCR
shRNA	small hairpin RNA
SI	syncytium-inducing
ssRNA+	single-stranded, positive-sense RNA
ssRNA−	single-stranded, negative-sense RNA
TAg	T antigen
Tc	cytotoxic T cells
TNF	tumor necrosis factor
TNFR-1	TNF receptor 1
TNM	tumor-node-metastasis
TRADD	TNF receptor-associated death domain
TUNEL	terminal deoxynucleotidyl transferase dUTP nick end labeling
UPR	unfolded protein response
US	unique short kinase
XBP1	X-box binding protein 1
ZIKV	Zika virus
